# Electrical stimulation enhances sciatic nerve regeneration using a silk-based conductive scaffold beyond traditional nerve guide conduits

**DOI:** 10.1038/s41598-024-65286-9

**Published:** 2024-07-02

**Authors:** Alireza Soltani Khaboushan, Ashkan Azimzadeh, Saman Behboodi Tanourlouee, Melina Mamdoohi, Abdol-Mohammad Kajbafzadeh, Konstantin V. Slavin, Vafa Rahimi-Movaghar, Zahra Hassannejad

**Affiliations:** 1 Pediatric Urology and Regenerative Medicine Research Center, Gene, Cell and Tissue Research Institute, Children’s Medical Center, Tehran University of Medical Sciences, No. 62, Dr. Gharib’s Street, Keshavarz Boulevard, Tehran, 1419733151 Iran; 2 Students’ Scientific Research Center, Tehran University of Medical Sciences, Tehran, Iran; 3 Department of Biomedical Engineering, Amirkabir University of Technology (Tehran Polytechnic), Tehran, Iran; 4 Department of Neurosurgery, University of Illinois at Chicago, Chicago, IL USA; 5 Sina Trauma and Surgery Research Center, Sina Hospital, Tehran University of Medical Sciences, Hassan-Abad Square, Imam Khomeini Ave., Tehran, 11365-3876 Iran

**Keywords:** Nerve repair, Conductive conduit, Stem cells, Electrical stimulation, Regeneration, Neurology, Preclinical research, Tissue engineering, Biomaterials - cells, Implants

## Abstract

Despite recent advancements in peripheral nerve regeneration, the creation of nerve conduits with chemical and physical cues to enhance glial cell function and support axonal growth remains challenging. This study aimed to assess the impact of electrical stimulation (ES) using a conductive nerve conduit on sciatic nerve regeneration in a rat model with transection injury. The study involved the fabrication of conductive nerve conduits using silk fibroin and Au nanoparticles (AuNPs). Collagen hydrogel loaded with green fluorescent protein (GFP)-positive adipose-derived mesenchymal stem cells (ADSCs) served as the filling for the conduit. Both conductive and non-conductive conduits were applied with and without ES in rat models. Locomotor recovery was assessed using walking track analysis. Histological evaluations were performed using H&E, luxol fast blue staining and immunohistochemistry. Moreover, TEM analysis was conducted to distinguish various ultrastructural aspects of sciatic tissue. In the ES + conductive conduit group, higher S100 (*p* < 0.0001) and neurofilament (*p* < 0.001) expression was seen after 6 weeks. Ultrastructural evaluations showed that conductive scaffolds with ES minimized Wallerian degeneration. Furthermore, the conductive conduit with ES group demonstrated significantly increased myelin sheet thickness and decreased G. ratio compared to the autograft. Immunofluorescent images confirmed the presence of GFP-positive ADSCs by the 6th week. Locomotor recovery assessments revealed improved function in the conductive conduit with ES group compared to the control group and groups without ES. These results show that a Silk/AuNPs conduit filled with ADSC-seeded collagen hydrogel can function as a nerve conduit, aiding in the restoration of substantial gaps in the sciatic nerve with ES. Histological and locomotor evaluations indicated that ES had a greater impact on functional recovery compared to using a conductive conduit alone, although the use of conductive conduits did enhance the effects of ES.

## Introduction

Peripheral nerve injuries (PNIs) are debilitating events that remarkably diminish the quality of life and impose a huge burden on the health care system and patients. PNI occurs at a rate of about 2.2% of trauma cases and is more common in males. Various causes lead to PNI, of which motor vehicle accident serves as the most prominent etiology^1–4^. The radial nerve is the most frequently injured in the upper limb, while the sciatic sustains the most damage in the lower limb^5^. In addition to the loss of motor function and subsequent muscle paralysis, incomplete repair leads to complications such as permanent neuropathic pain and the need for intensive care, diminishing the patient’s quality of life^3,6,7^.

The neurosurgical approach for PNI is end-to-end neurorrhaphy or sutureless repair using fibrin glue unless there is a large gap necessitating the application of grafts or nerve conduits. Autografts are usually the clinical gold standard for transected nerves with large gaps^8,9^. Although auto-transplantation provides the best outcome, the functional recovery of the peripheral nerve and muscles is still incomplete; this is primarily because the axons regenerate slowly, and the peripheral nerves and glia only maintain their regenerative capacity for a short period. Moreover, sources for auto-transplantation are limited, and it usually incorporates damage to another part of the body. Similar to auto-transplantation, nerve transfer provides suitable outcomes for peripheral nerve repair, but it also leads to donor-side morbidity^8^.

To overcome the problems auto-transplantation faces, various types of scaffolds, including nanofibrous scaffolds, multichannel, and conductive nerve conduits, have been studied^10–12^. Nerve conduits are structures that guide the nerves to grow in a specific direction to repair large gaps and should have some characteristics, including flexibility, biocompatibility, biodegradability, mechanical stability, and permeability to nutrients and oxygen. Electrical conductivity is another suitable feature of the nerve conduits^7^. Researchers have also explored different methods for creating nerve conduits to improve nerve regeneration and guide axons to the appropriate distal stumps. Some of these methods include incorporating microgrooves into the interior surface of the conduit, filling the lumen with hydrogels, or using tissue-derived extracellular matrix (ECM)^13–17^.

Natural, synthetic, or blended biomaterials have been used for designing nerve conduits^12^. Whether used alone or in combination with synthetic materials, silk has been proven to be a suitable natural-based biomaterial for nerve conduits. It has been recognized as an exceptional biomaterial with advantageous properties for engineering nervous tissue, as indicated by numerous studies^18–20^. Silk constructs are permeable to water and oxygen, allowing a suitable nutrient supply for the tissue. In addition, there have been reports that silk enhances cell attachment and proliferation. Also, it has low immunogenicity and, due to mechanical strength, prevents the collapse of the conduit^21,22^.

Silk has been made conductive for biomedical applications using various methods. This includes coating or polymerization of conductive polymers like PPy, PANI, PEDOT, and their copolymers^23^. In addition, materials such as graphene oxide, carbon nanotubes, and gold nanoparticles (AuNPs) have been incorporated into the silk. It has been shown that using AuNPs for creating conductive scaffolds can enhance Schwann cell proliferation^24,25^.

Electrical stimulation (ES) has been reported as an effective intervention for improving nerve regeneration. In healthy tissues, electric potential gradients are usually small. However, after injury, these gradients increase due to breached epithelial barriers, which lead to the formation of electric currents that flow towards the compromised epithelium, establishing lateral electrical fields. These electrical fields are believed to play a role in controlling and integrating various cell behaviors such as proliferation, division, migration, and nerve sprouting. Exogenous ES attempts to mimic these endogenous electric fields and has been shown to promote nerve regeneration. It has been reported that applying the ES on end-to-end coaptation of the nerves or even auto-transplantation could improve the result of nerve regeneration^26,27^. ES can stimulate Schwann cell proliferation, neural cell differentiation, axonal growth and extension, and the production of neurotrophic factors^28,29^. It influences cellular polarization, ionic currents across the cell membrane, gene expression, and the release of growth-promoting molecules^30^. ES has also been found to accelerate neuronal expression of cytoskeletal proteins and growth-associated proteins that are important for neuronal formation and repair^31^.

In this study, we aimed to evaluate the effect of a conductive nerve conduit and ES on the regeneration of the sciatic nerve in a rat model with transected injury. To achieve this, we fabricated a conductive conduit using silk fibroin (SF) and AuNPs. In addition to determining the physicochemical characteristics of the conductive nerve conduit, we evaluated two different fillings, collagen hydrogel and decellularized sciatic nerve tissue, based on their microstructure and cell metabolic activity. Moreover, our research explores different treatment approaches to validate the effectiveness of the conductive nerve conduit, ES, and combination therapy in promoting nerve regeneration and functional recovery.

## Materials and methods

### Isolation and identification of adipose-derived mesenchymal stem cells

Adipose-derived mesenchymal stem cells (ADSCs) were extracted from transgenic rats that expressed green fluorescent protein (GFP). Adult female Wistar rats were euthanized with ketamine/xylazine overdose, and adipose tissue was collected from the inguinal region. The adipose tissues were washed thrice with phosphate buffered saline (PBS) to remove debris and red blood cells and cut into small pieces. The obtained tissue explants were cultured in DMEM/F-12 (Gibco™, USA) supplemented with 10% fetal bovine serum (FBS, Gibco™, USA) and 2% penicillin/streptomycin (Sigma, Germany) and incubated at 37 °C and 5% CO_2_ until the outgrowing cell confluence reached 80%. Flow cytometry was used to assess cell surface markers such as CD34, CD90, and CD105 to confirm the lineage of the extracted mesenchymal cells. The cells were used between passages 3–5, and 5 × 10^5^ cells were seeded within each conduit the day before implantation using collagen hydrogel as the conduit filling.

### Silk fibroin solution preparation

SF solution was prepared based on the literature^32^. Small pieces of Bombyx mori silk cocoons (Iran silk research center, Gilan, Iran) were boiled in 0.02 M Na_2_CO_3_ (Merck, Germany) aqueous solution for 30 min. The resulting fibers were rinsed three times with deionized water to remove sericin protein. After drying overnight at room temperature, the SF fibers were dissolved in 9.3 M LiBr (Sigma-Aldrich, Germany) (1 g of dried fibers in 4 mL of LiBr solution) at 65 °C for 4 h, resulting in a viscous solution. The solution was then dialyzed in a cellulose tube (12400 Dalton, Sigma) against distilled water for 3 days at room temperature to remove residual salts. The SF solution was centrifuged twice at 11,000 RPM to remove insoluble particulates, resulting in a final concentration of 7–8% (w/v), which was used without dilution. The solution was stored at 4 °C and used within 2 weeks.

### Synthesis of gold nanoparticles (AuNPs)

The synthesis of colloidal Au was carried out according to the literature^33^. Briefly, a solution of hydrogen tetrachloroaurate trihydrate (HAuCl_4_.3H_2_O, Merck, Germany) (1 mM) in a round bottom flask was boiled. Trisodium citrate solution (Na_3_C_6_H_5_O_7_, Merck, Germany) (38.8 mM) was added rapidly to the boiling solution under vigorous stirring. The solution was stirred continuously for about 5 min and underwent a series of color changes before turning red. The suspension was stored at 4 °C until needed. The maximum optical absorbance was found at λ_max_ ≈ 520 nm.

### Preparation of SF/AuNPs films

First, the pH of the gold suspension was adjusted to 7.5 using NaOH (Merck, Germany) solution. The suspension was then added to the SF solution with a volume ratio of 1:2 silk: AuNPs and stirred gently for 10 min to ensure a homogeneous solution. Next, poly (ethylene oxide) (PEO, MW = 900,000 g/mol, Sigma-Aldrich, Germany) (0.16 wt/vol. %) was added to the SF/AuNPs solution. The final solution was stirred gently at room temperature and cast onto a 6-well culture plate. 100 µm of solution was cast in each plate to obtain a 41.27 ± 4.15 µm film thickness. After the scaffold was air dried, β-sheet formation was induced by treating it with 90% aqueous methanol (Merck, Germany) solution for 30 min. In order to create non-conductive conduits, deionized water was replaced by AuNPs suspension. Finally, the PEO was removed from the prepared film by immersing it in a water bath at room temperature for 24 h. The films were sterilized using 70% EtOH for in vitro and in vivo assessments.

### Preparation of collagen hydrogel and decellularized rat sciatic tissue as conduit filler

Type I collagen was extracted from the rat tail tendon, as previously approved^34^. Briefly, the collected collagen fibers were placed in acetone (C_3_H_6_O, Merck, Germany) for 5 min and then transferred to isopropanol 70% (C_3_H_8_O, Merck, Germany) for 5 min. Eventually, after the fibers were dissolved in acetic acid for 48 h, a viscous solution was obtained. Finally, the solution was frozen at -20 °C and freeze-dried to get collagen sponges. The prepared collagen sponges were dissolved in 0.1 M acetic acid at a final 6.25 mg/ml concentration. The resulting solution was combined with a double buffer system (1.3M NaCl and 0.2M Na_2_HPO_4_.12H_2_O), 0.3M NaOH solution, and 10 × DMEM-F12 on ice to keep the temperature at 4 °C. In one experiment, 1 ml of collagen solution was mixed with 100 µl buffer, 300 µl NaOH, and 100 µl 10 × DMEM-F12. If seeding cells within the collagen hydrogel, the 10 × DMEM-F12 was replaced with cell suspension. Finally, the solution was incubated at 37 °C for 40 min to form the collagen hydrogel.

Decellularization of sciatic tissue was conducted in a modified protocol^35^. Briefly, an adult Wistar rat was euthanized using a ketamine/xylazine overdose, and the sciatic nerve tissues were harvested under aseptic conditions and pooled in sterilized PBS. The harvested tissues underwent three washes with PBS. The tissues were stirred in 1% sodium dodecyl sulfate (SDS) solution in deionized water at 25 °C for 24 h to facilitate decellularization. After SDS treatment, the tissue was washed in deionized water three times, each for 30 min. In the next step, tissues were stirred in 1% Triton X-100 (Merck, Germany) aqueous solution for one hour, followed by several washes over 72 h. Finally, the decellularized tissue was incubated in 70% ethanol for 5 min, treated with a cocktail of antibiotics containing penicillin, cefazolin, and amphotericin for 20 min, and washed several times with sterilized PBS^36^. The efficacy of the collagen hydrogel and decellularized sciatic nerve on enhancement of ADSCs proliferation was assessed using MTT assay.

### Fabrication of nerve conduit filled by collagen hydrogel

The SF/AuNPs films were shaped as conduits to support topography and promote the growth of peripheral nerves. First, the films were wrapped around a glass rod with an outer diameter of 1.8 mm. A 4% SF solution was used to stabilize the conduit. Finally, the prepared conduits were filled with collagen hydrogel containing ADSCs and incubated at 37 °C and 5% CO_2_.

### Physicochemical characterization of SF/AuNPs films

#### X-ray diffraction analysis

The XRD scans of the samples were recorded using the X-ray diffractometer (Inel EQUINOX3000, France) operating at 40 kV and 30 mA. The samples were scanned in the 2θ range of 5°–80° with a scanning speed of 10°/min.

#### Electrical conductivity

The electrical resistances of SF/AuNPs and SF films were measured repeatedly using a multi-meter by the four-probe method under ambient conditions. After three measurements, conductivity was calculated using the following formula:

1 $${\text{R}} = \uprho {\text{L}}/{\text{Wt}}$$

2 $$\upsigma = {1}/\uprho$$

where R, ρ, L, W, and t are the slope of the current flow-voltage curve, resistivity, length, width, and thickness of the scaffold, respectively, and σ is the electrical conductivity of the scaffolds.

#### Mechanical properties of SF/AuNPs films

The scaffold’s tensile properties were assessed using a uniaxial testing instrument (Instron 5566, USA). Samples were cut to 15 mm × 5 mm × 0.03 mm (height × width × thickness), and loaded into tension clamps with an initial gauge length of 10 mm. Prior to testing, all samples were stored in a PBS bath at room temperature. The samples were then elongated at a 3 mm/min rate until failure. Stress/strain plots were used to calculate the initial elastic modulus (EM), ultimate tensile strength (UTS), burst strength, and % elongation to failure (ETF). The EM was estimated through a least-squares (LS) fit within the linear elastic region, while the UTS was determined as the highest stress value achieved during the test. The ETF was the last data point before rupture. At least three samples were used to calculate the average modulus with standard deviation.

#### Field emission scanning electron microscopy (FE-SEM)/energy dispersive X-ray analysis (EDXS)

Imaging was conducted with FE-SEM ZEISS (Sigma 300, Germany) to investigate the scaffold morphology. A 40 nm coating of gold was sputter-coated onto the sample. ImageJ software was used to analyze the acquired images. The elemental content identification and quantification were assessed using the energy-dispersive X-ray spectrometer (EDS, TESCAN MIRA3). Integrated mapping software was then used to examine the distribution of the elements.

### MTT assay

The effect of AuNPs, decellularized sciatic tissue, collagen hydrogel, SF, and SF/AuNPs films on cellular metabolism was evaluated through MTT assay using rat ADSCs. The cells in plates without scaffolds were used as controls. Each scaffold was placed in a well of a 96-well tissue-culture plate. A total of 1 × 10^4^ cells were seeded onto the scaffolds in each well and incubated at 37 °C, 5% CO_2_ for 48 h. After that, 10 μL of MTT solution with a concentration of 5 mg/ml was added to each well. After 4 h of incubation at 37 °C, the medium was replaced with 100 µl DMSO, and absorbance was measured at 540 nm using a microplate reader (Elx800, BioTek, USA). All MTT results are obtained from two different experiments with 7 replications for each sample.

### Analyzing the distribution of ADSCs within collagen hydrogel and on SF/AuNPs films

The ADSCs were obtained from GFP transgenic rats. Forty-eight hours after cell seeding, the scaffolds were fixed in 4% PFA and washed in phosphate-buffered saline (PBS). We then captured digital images of the scaffolds using a fluorescence microscope. To ensure that the cells visible in the images were within the collagen hydrogel, we transferred the hydrogel-containing cells to a new cell culture plate before imaging.

### Experimental design

Adult female Wistar rats (200–250 g, 12 weeks old) were housed under controlled temperature and humidity with a 12:12 light: dark cycle and fed standard chow and water ad libitum. 25 rats were randomly divided into five groups: (1) gold standard control (n = 5), (2) non-conductive conduit without ES (n = 5), (3) conductive conduit without ES (n = 5), (4) non-conductive conduit with ES (n = 5), and (5) conductive conduit with ES (n = 5) (Table 1).

**Table 1:** Experimental design.

No.	Code of animal groups	Definition of animal groups	No. of animals
1	Control	Gold standard (control)	5
2	Non-conductive − ES	Non-conductive conduit without ES	5
3	Conductive − ES	Conductive conduit without ES	5
4	Non-conductive + ES	Non-conductive conduit with ES	5
5	Conductive + ES	Conductive conduit with ES	5

### Animal surgery and electrical stimulation application

**Figure 1:**
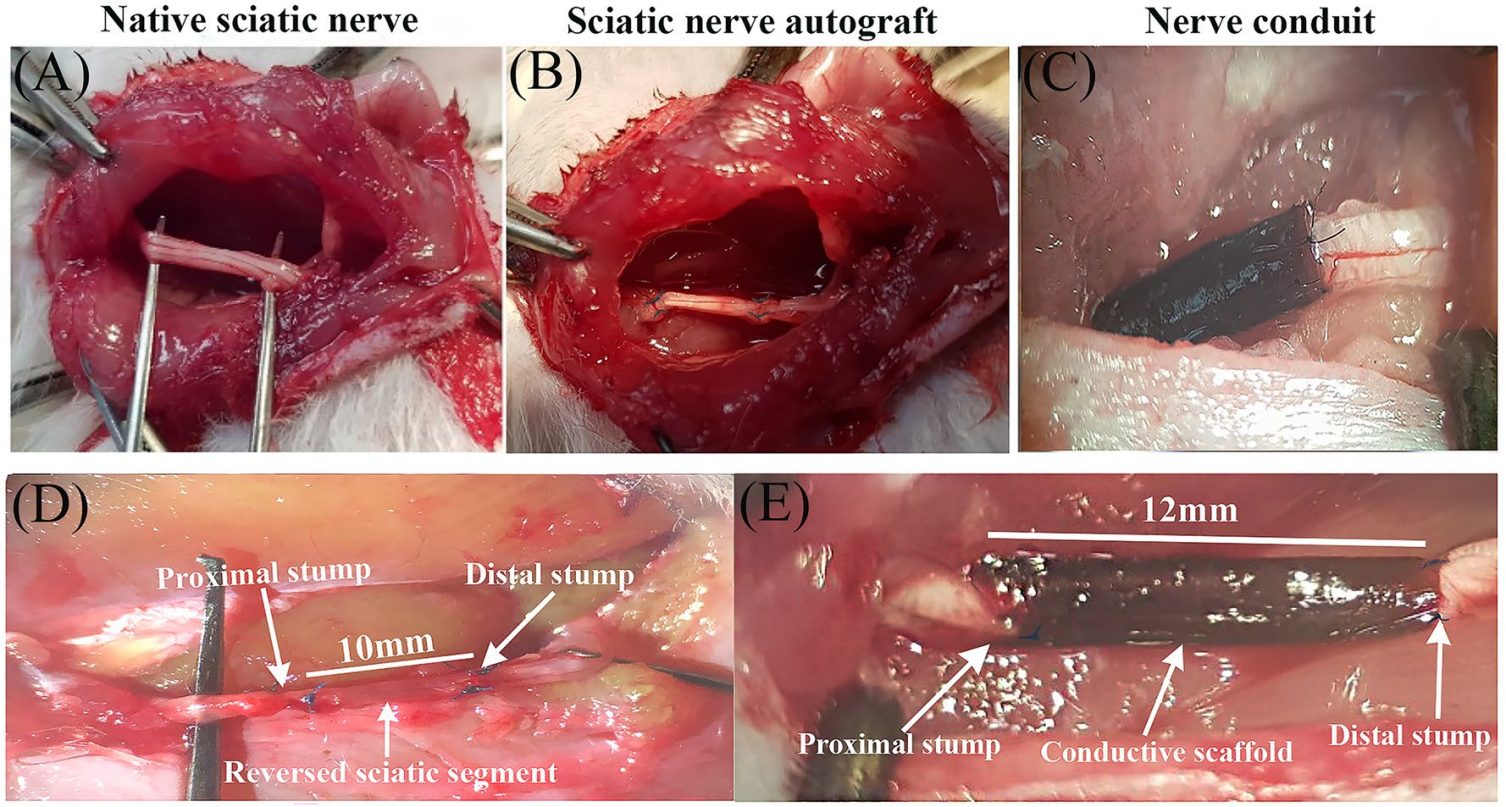
Placement and securing the autograft and nerve conduits following sciatic nerve transection. (**A**) Native sciatic nerve before transection, (**B** and **D**) Sciatic nerve autograft, in this group the dissected segment of the sciatic nerve was reversed in polarity and microsurgically coapted distally and proximally to the proximal and distal stumps, respectively. (**C** and **E**) In the conduit groups, a 12 mm conduit was placed deep in the gap, and one mm of each stump was inserted into the conduit to prevent the staggering of the nerve.

The surgical procedures were conducted using an operating microscope and an aseptic approach in the animal facility room. Rats were anesthetized with an intraperitoneal injection of a mixture of Ketamine (90 mg/kg) and Xylazine (10 mg/kg). The animals' body temperature was closely monitored and placed on an electric heating pad during surgery. A 2 cm incision was made parallel and just below the right femur bone of the rats. The septum separating the vastus lateralis and biceps femoris muscles was bluntly dissected to reveal the sciatic nerve. The sciatic nerve was delicately extracted from the adjacent tissues. A 10 mm segment of the right sciatic nerve was transected in all animals 5 mm proximal to its bifurcation. In control group, the dissected segment of the sciatic nerve was reversed in polarity and microsurgically coapted distally and proximally to proximal and distal stumps, respectively, using two epineurial 10–0 nylon monofilament sutures (Ethicon Inc., Somerville, NJ). In other groups, a 12 mm conduit was placed deep in the gap, and one mm of each stump was inserted into the conduit to prevent the staggering of the nerve; the conduit was secured to both sides using two epineurial 10–0 nylon sutures (Fig. 1). After securing the conduit for groups 4 and 5 to deliver ES, the cathode (+) electrode was 5 mm proximal to proximal coaptation, and the anode (−) electrode was placed to the approximate biceps muscle. Thereafter, ES was delivered with 20 Hz frequency, 0.1 ms duration of the pulse, and 1.2 V (the maximum voltage tolerated by the animal) for 30 min. After ES delivery, electrodes were removed, and muscle approximation and skin closure were done via 5–0 Vicryl® suture (Ethicon Inc., Somerville, NJ). To prevent autotomy, saturated picric acid was topically applied to the hindlimb^37^.

### Walking track analysis

SFI was the most frequent index in evaluating hindlimb motor function following sciatic nerve transection^38–40^. In addition, static sciatic index (SSI), peroneal functional index (PFI), and tibial functional index (TFI) were calculated and reported^40–42^.

#### Sciatic functional index (SFI)

The rats were placed in a corridor with a length of 1.5 m and a width of 20 cm with a height of 40 cm covered thoroughly with see-through glass. The background of the corridor was covered with black paper to increase the contrast, remove excessive lights, and prevent reflexes. A mirror with a slope of 45˚ was placed below the gallery to see the reflection of the paw trace of the rats. Rats were trained to walk through the corridor one week before surgery. Each rat walked through the corridor thrice and was filmed during the whole process. Recorded videos were analyzed, and 5 captures were used to calculate SFI for each rat. SFI compares geometric measures of the injured paw with the normal paw. SFI could be calculated through the following formula^38–40^:

$$SFI = - 38.3 \times \left( {\frac{EPL - NPL}{{NPL}}} \right) + 109.5 \times \left( {\frac{ETS - NTS}{{NTS}}} \right) + 13.3 \times \left( {\frac{EIT - NIT}{{NIT}}} \right) - 8.8$$

where EPL, ETS, and EIT are experimental print length, experimental toe spread, and experimental intermediary toe spread, respectively, and NPL, NTS, and NIT are the same indices for the normal paw.

#### Static sciatic index (SSI)

SSI is a time-saving method for the evaluation of peripheral nerve regeneration in rats. It only considers static factors and ignores print length. It is calculated according to the following equation^42^:

$$SSI = 108.44 \times \left( {\frac{ETS - NTS}{{NTS}}} \right) + 31.85 \times \left( {\frac{EIT - NIT}{{NIT}}} \right) - 5.49$$

ETS, NTS, EIT, and NIT are explained in the SFI section.

### Histological evaluations

Sciatic nerve tissues were harvested, fixed in 10% neutral-buffered formalin, and processed for paraffin embedding. Sections of 5 µm were stained with Hematoxylin and Eosin (H&E) for general tissue morphology, where hematoxylin imparted a blue hue to the nuclei while eosin stained the cytoplasm pink. For myelin detection, sections underwent Luxol Fast Blue (LFB) staining overnight. Subsequently, immunohistochemistry was performed on deparaffinized sections, involving heat-induced antigen retrieval, blocking steps, and overnight incubation with primary antibodies specific to neurofilament (NF200, ab7255) and S100 (ab34686) proteins. Detection involved biotinylated secondary antibodies, streptavidin-peroxidase complex, and chromogen development with 3,3'-diaminobenzidine (DAB, Abcam), followed by hematoxylin counterstaining. The stained sections, indicative of various cellular and tissue elements, were systematically analyzed under a bright-field microscope, with images captured for detailed qualitative and quantitative assessment of histological characteristics and specific protein expression patterns^43^. The samples from all groups at 6 weeks were stained with 4,6-diamidino-2-phenylindole (DAPI) to mark the nucleus of all cells. Moreover, the ADSCs seeded on the scaffold were GFP positive, and immunofluorescent microscopy was used to capture the survival, migration, and distribution of GFP positive ADSCs and rat cells.

### Transmission electron microscopy (TEM) of tissues

Sciatic nerves were surgically extracted from adult rats and were immediately fixed in 2.5% glutaraldehyde with 0.1M cacodylate buffer, followed by post-fixation in 1% osmium tetroxide, ensuring the preservation of cellular ultrastructure. The samples underwent a graded dehydration process in an ethanol series before embedding in epoxy resin. After polymerization, ultrathin sections (60 nm) were sliced using an ultramicrotome and collected on copper grids for imaging. These procedures aimed to maintain the intricate details of the sciatic nerve, including Büngner bands, Schwann cells, axons, and histiocytes, for high-resolution visualization under the TEM.

The TEM analysis was conducted using a Philips CM200 Transmission Electron Microscope featuring a field emission gun and a 0.24 nm resolution. It includes a Gatan Orius SC1000 CCD camera for improved image capture and operates at 80–200 kV accelerating voltages. Initial low-magnification examination identified regions of interest, which were subsequently imaged at higher resolution. The resulting greyscale micrographs were digitally enhanced for clarity and contrast before undergoing a pseudo-coloring process. This coloring was not indicative of inherent staining properties. Still, it was applied manually to distinguish various structural aspects: Büngner bands (red), Schwann cell nuclei (blue), Schwann cell cytoplasm (yellow), axons (purple), and histiocytes (green). This meticulous process with expert verification allowed for a more intuitive visual interpretation of the complex cellular interactions within the sciatic nerve tissue. However, it did not provide molecular-level specificity^44^.

### Image analysis

Image analysis was done using NIH-ImageJ version 1.53 k (https://imagej.net/ij/download.html). The evaluations encompassed counting myelinated axons in the nerve sections, gauging the myelin sheath thickness from TEM images, and quantifying the IHC results based on the percentage of positively stained areas. For a thorough assessment, three random fields of 80 mm × 60 mm each from every nerve specimen were inspected at 400× magnification^45^.

### Statistical analyses

All quantitative data are expressed as mean ± standard deviation (SD). Statistical comparisons for histopathological findings were performed by one-way analysis of variance (ANOVA) using GraphPad Prism version 8.0.2. Moreover, one-way ANOVA was used to compare the SFI scores between groups, and a linear mixed model with Tukey posthoc test was used to compare the SFI scores between rats through time points using R programming language version 4.2.1. P values of less than 0.05 were considered statistically significant.

### Ethical approval

All animal experiments were performed in accordance with Tehran University of Medical Sciences (TUMS) guidelines, and animal care protocols were approved by the Ethical Committee of the TUMS (IR.TUMS.CHMC.REC.1398.120). Additionally, reporting of use of experimental animals in this study adhered to the recommendations outlined in the ARRIVE guidelines.

## Results

### Flow cytometry

The results of the flow cytometry analysis indicated that the cells extracted from adipose tissue expressed mesenchymal stem cell markers CD90 (86.3%) and CD105 (98.5%) while showing a lack of expression of hematopoietic marker CD34 (2.27%). Based on previous reports a positive expression of CD105 and CD90 above 85%, along with a negative expression of CD34 below 5%, confirms that the extracted cells are mesenchymal stem cells and not hematopoietic stem cells^46^.

### Characterization of acellular rat sciatic nerve and collagen hydrogel

**Figure 2:**
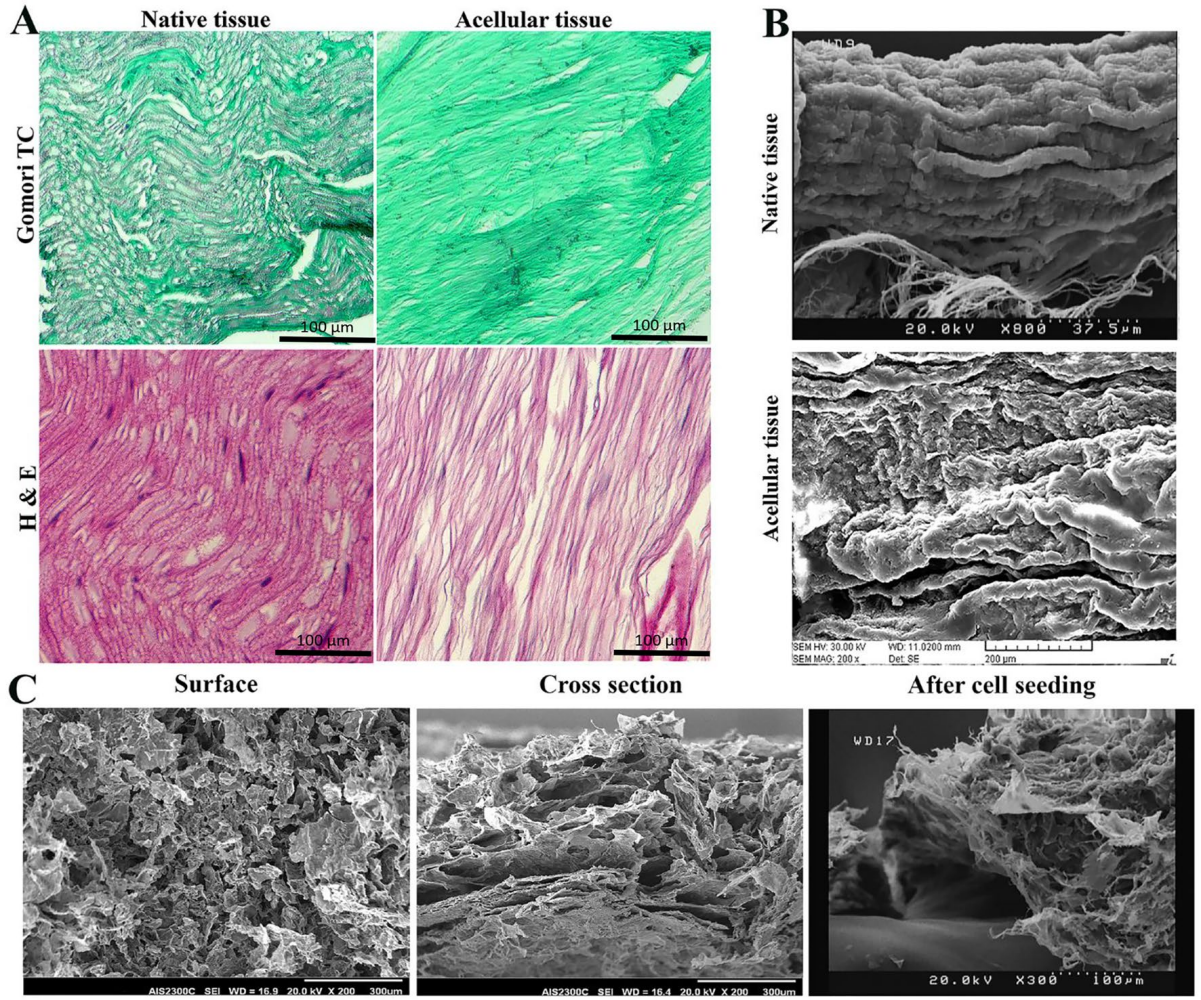
Structural and histological assessments of acellular rat sciatic nerve and collagen hydrogel. (**A**) The rat sciatic nerve tissue was histologically evaluated in its native and decellularized states. Hematoxylin and Eosin (H&E) staining demonstrated the complete removal of the genetic component while retaining the extracellular matrix of the tissue. Additionally, Gomori trichrome staining was utilized to assess the preservation of collagen in the tissue's extracellular matrix during decellularization. (**B**) SEM images of native and decellularized rat sciatic nerve. (**C**) SEM images showing the surface and cross section of collagen hydrogel both prior and after seeding with adipose-derived stem cells.

The efficacy of the decellularization process on the sciatic nerve tissue was assessed using H&E and Gomori trichrome staining. Figure 2A demonstrates that the decellularization protocol effectively removed all cell nuclei while retaining the extracellular components, particularly collagen fibers, as evidenced by Gomori trichrome staining. Moreover, the examination of SEM images demonstrates the effective preservation of the sciatic nerve's fibrous structure through the decellularization process (Fig. 2B). Additionally, a comparison between the microstructure of the decellularized sciatic nerve tissue and collagen hydrogel was conducted using SEM imaging. Notably, Fig. 2B and C illustrate that collagen hydrogel exhibits larger pore size and increased porosity, enabling enhanced cell migration and proliferation within its framework.

### Characterization of SF/AuNPs films

**Figure 3:**
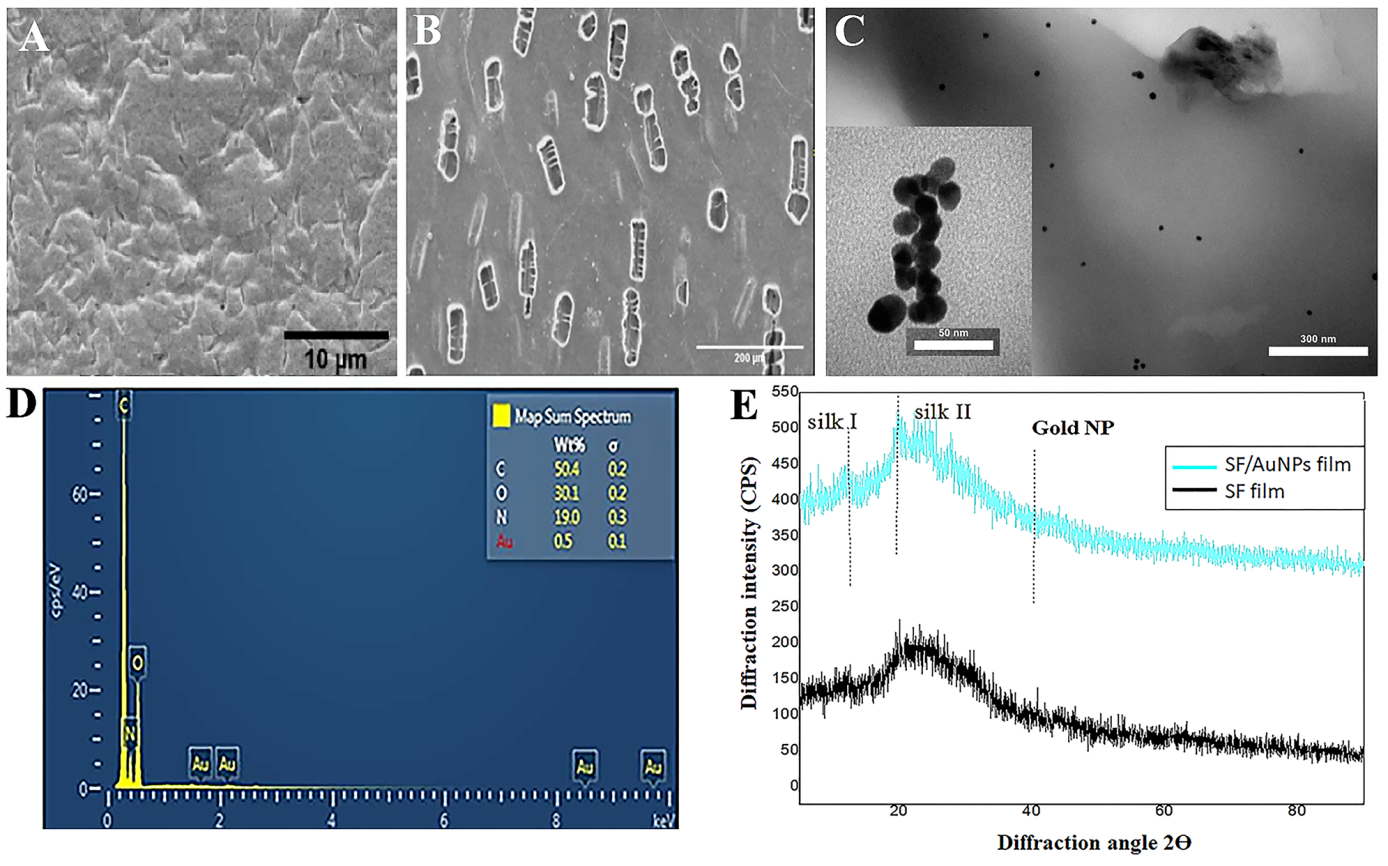
Characterization of silk fibroin (SF) films containing gold nanoparticles (AuNPs) (**A**) FE-SEM images showing the surface and (**B**) cross-section morphologies of the SF film after washing out the PEO. (**C**) TEM images of AuNP with the average diameter of 16 ± 2.5 nm showing the morphology (insert) and distribution of nanoparticles within the SF/AuNPs films (**D**) EDAX analysis of SF/AuNPs films, showing peaks of C, N, O that respect to fibroin protein and Au was for gold ions in the films. (**E**) XRD patterns of SF films and SF/AuNPs films. The grey dotted line indicates the gold reflection at 38, silk I at 12.3°, and silk II at 19.9°.

The morphology of the surface and cross section of SF films after PEO extraction are shown in Fig. 3A and B, respectively. After PEO leaching, a rough topography was observed on the surface of SF films and rectangular pores in the cross section area, which can be attributed to PEO micro-phase separation. Furthermore, TEM images showed a uniform distribution of AuNPs within the SF films with an average diameter of 16 ± 2.5 nm (Fig. 3C). Also, the surface of the SF/AuNPs films was evaluated by energy dispersive X-ray analysis (EDAX), as shown in Fig. 3D. The graph exhibited peaks of C, N, and O regarding fibroin protein and Au for gold ions in films.

Moreover, XRD scans of the SF/AuNPs films were performed to investigate the crystalline structure of the films. The peak at 19.9° is characteristic of the silk II structure and confirms the existence of β-sheet structures; 12.3° represents silk I in these films. Furthermore, XRD confirmed the presence of AuNPs with an increasing diffraction peak at 38° in the SF/AuNPs films and indicated the presence of pure AuNPs in the films. In addition, the silk fibroin diffraction peaks were preserved regardless of the presence of the AuNPs. The demonstrated peaks at a scattering angle (2θ) of about 38° can be assigned to the (111) Bragg’s reflections of the cubic structure of metallic gold. The XRD results exhibited that the resultant particles had face-centered cubic (FCC) structures of metallic AuNPs. A comparison of the crystalline structure of the SF and SF-AuNPs films after methanol treatment shows that SF/AuNPs have more β-sheet content than SF film, which is due to the presence of AuNPs in the film structure and the effect of these nanoparticles on the formation of crystalline structures in the films (Fig. 3E).

Tensile testing of scaffolds prior to implantation was assessed (Table 2). The films exhibited suitable ultimate tensile strength to support the peripheral nerve. Moreover, the film exhibited a significantly higher EM value, measuring 37.7 times greater than that of the rat's normal sciatic nerve, which effectively facilitates the suturing process of the conduit during surgical procedures. Also, the films demonstrated a lower elongation to failure and burst strength than the rat nerve, but according to other studies, these amounts are appropriate for nerve tissue engineering applications.

**Table 2:** Comparison of mechanical properties of SF-AuNPs film and fresh rat sciatic nerve.

Samples	Ultimate tensile strength (Mpa)	Ultimate tensile strain (mm/mm)	Elastic modulus (Mpa)	Elongation to failure (%)	Burst strength (N)
SF/AuNPs film	1.62** ± **0.32	0.218** ± **0.04	21.90** ± **6.3	27.57** ± **11.8	0.458** ± **0.01
Rat Sciatic nerve	2.72 ± 970	0.810 ± 0.114	0.58 ± 150	77	2.14 ± 0.76

Moreover, electrical flow voltage curves were obtained for the SF and SF/AuNPs films using the four-point probe method. It was observed that within the voltage range measured, the electronic conduction of the SF/AuNPs films was 7.24 × 10^–3^ (S/cm), and the resistance of the SF/AuNPs films was measured to be 2.13 (ohm), while this was lower for the SF film (Table 3). Thus, it is considered that this study provides an appropriate SF/AuNPs film to acquire sufficient electrical conductivity for nerve tissue regeneration.

**Table 3:** Electrical conductivity of SF and SF/AuNPs films.

Scaffold	Resistance (ohm)	Volume conductivity (S/cm)
SF film	1.5 × 10^6^	6.6 × 10^–9^
SF-AuNPs film	2.13 × 10^5^	7.24 × 10^–8^

### MTT assay

**Figure 4:**
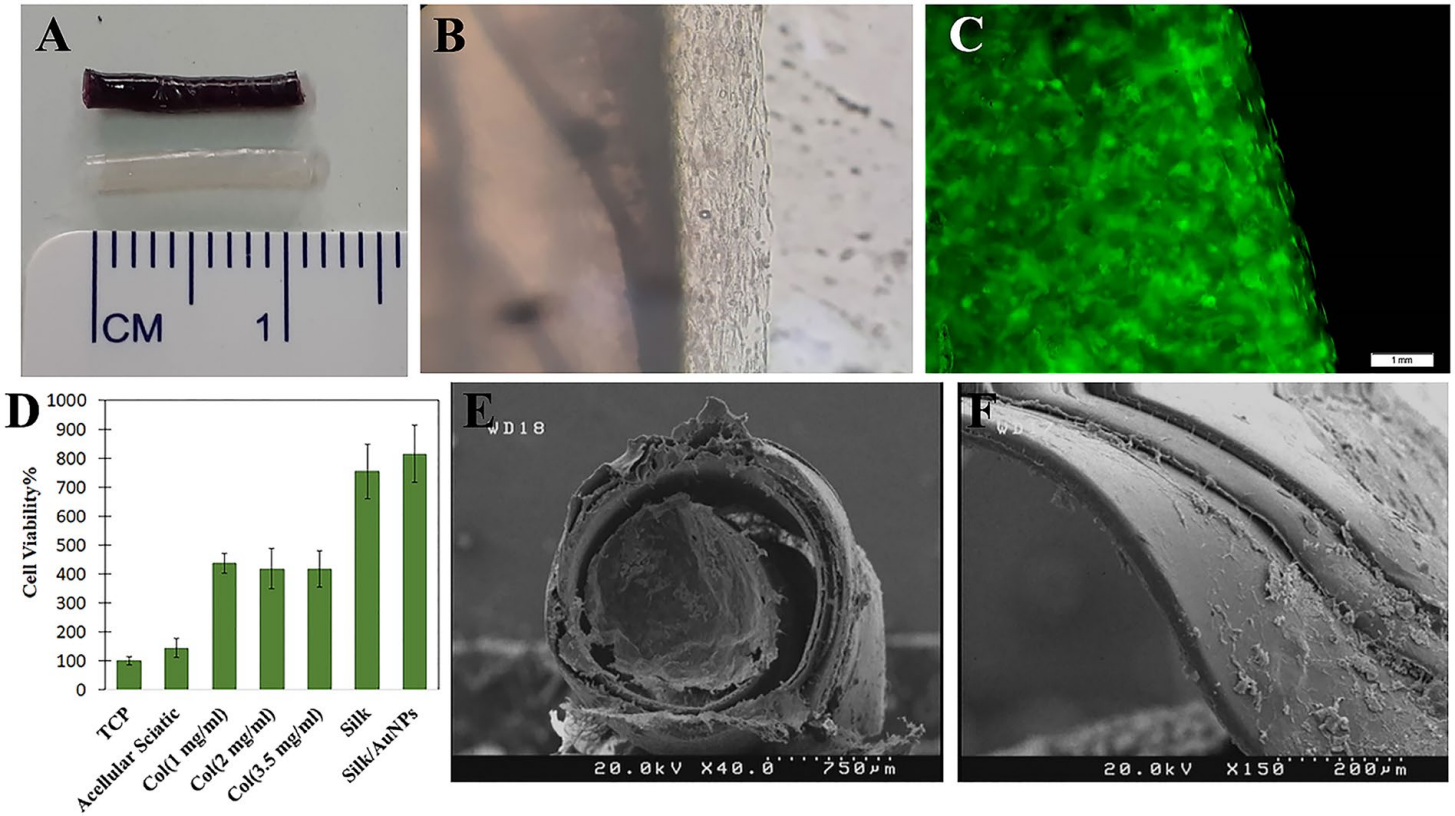
Nerve conduit structure and its effect on cell viability. (**A**) Dimensions of both conductive (upper) and non-conductive (lower) nerve conduits. (**B**) A photograph of a conductive conduit taken 24 h after cell seeding shows a thin layer of collagen hydrogel containing ADSCs around the nerve conduits, confirming the presence of cells inside and outside the conduit. (**C**) Fluorescent imaging of GFP positive ADSCs within the collagen hydrogel. (**D**) MTT assay result showing ADSCs proliferation on tissue culture plate (TCP), acellular sciatic nerve, collagen hydrogel with concentrations of 1, 2, and 3.5 mg/ml, silk film, and silk film containing AuNPs. (**E**) SEM image from the surface of the nerve conduit. (**F**) SEM image showing the cross section of the nerve conduit filled with collagen hydrogel. The thickness of the SF films was 41.27 ± 4.15 µm.

The proliferation of ADSCs on different substrates, including acellular sciatic nerve, collagen hydrogels with various concentrations of 1, 2, and 3.5 mg/ml, silk film, and silk film containing AuNPs was determined by the MTT assay in comparison to tissue culture plate as a control (Fig. 4D). Forty-eight hours after cell seeding, the proliferation was significantly higher within collagen hydrogels compared to the acellular sciatic nerve. Therefore, the nerve conduits were filled with collagen hydrogel for further evaluation. ADSC proliferation was also greater on silk films than on all other substrates, indicating that SF/AuNP films could provide a suitable substrate for transplanted and endogenous cells. The 3-dimensional environment provided by the scaffolds was identified as the main factor contributing to the increased proliferation of ADSCs. It was also observed that the decellularization process of the sciatic nerve might denature ECM proteins, as cell proliferation was more significant on pure collagen hydrogels compared to acellular sciatic nerve. Additionally, the surface roughness of silk membranes and conductivity of silk/AuNPs films were the most prominent factors affecting cell proliferation.

### Characterization of nerve conduit filled with collagen hydrogel

The dimensions of the fabricated nerve conduits are displayed in Fig. 4A. Figure 4B presents a photograph of a conductive conduit 24 h after cell seeding, demonstrating a thin layer of collagen hydrogel containing ADSCs around the nerve conduits, confirming the presence of cells both inside and outside the conduit. Additionally, fluorescent imaging of GFP positive ADSCs illustrates cell adhesion and uniform distribution within the collagen hydrogel, as shown in Fig. 4C. SEM image from the surface of the conduit and cross-section of the conduit filled with collagen hydrogel has been demonstrated in Fig. 4E and F, respectively.

### Animal surgery

Twenty five Wistar female rats with a mean weight of 227.31 ± 30.14 underwent sciatic transection surgery. Autografts served as controls, and 12 mm conductive or non-conductive conduits comprised other groups with or without ES. All animals tolerated surgery. Picric acid and critical observation were used to prevent autotomy of the hindlimb, which was successful.

### Walking track analysis

#### SFI

**Figure 5:**
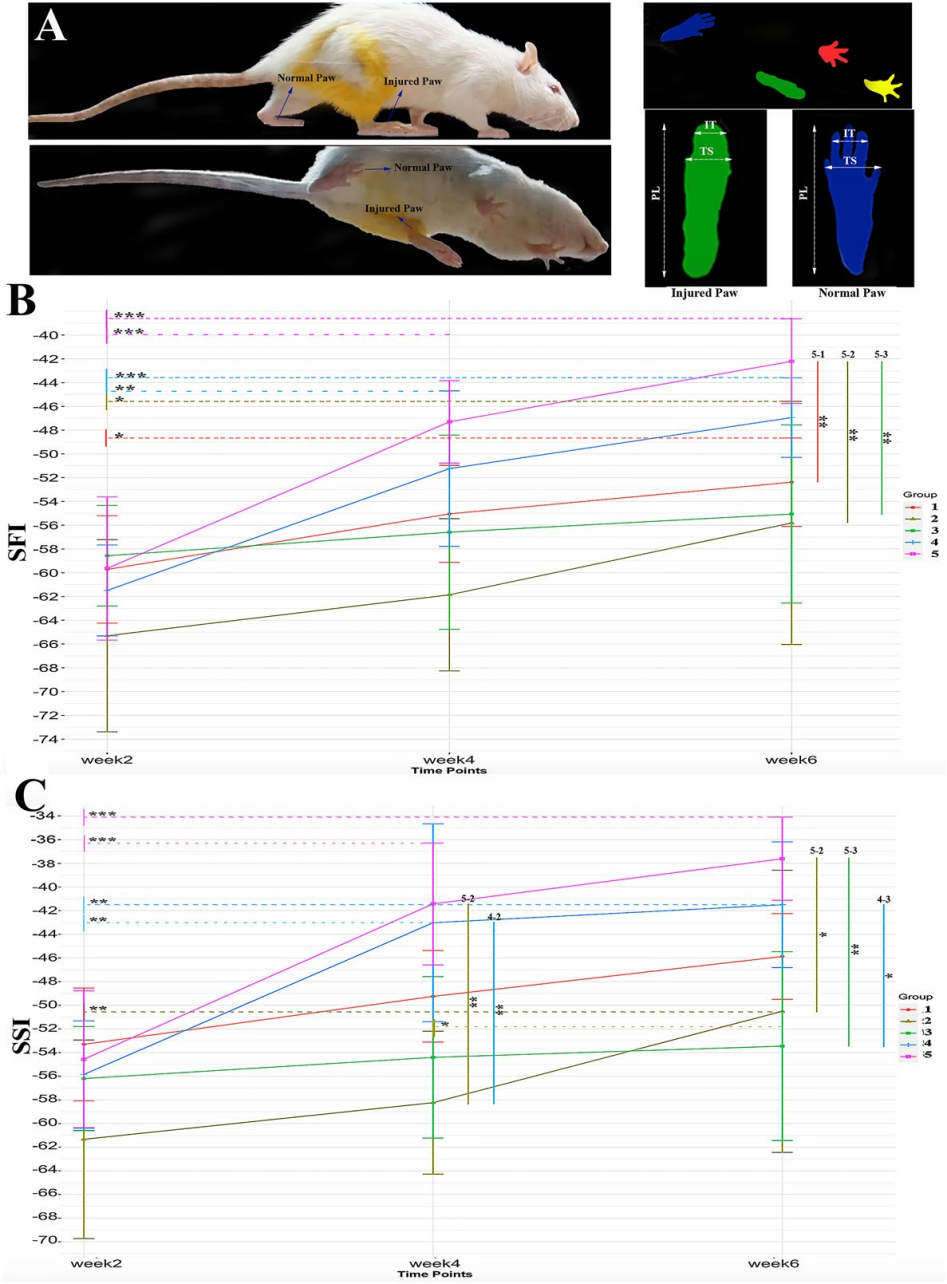
Locomotor assessments. (**A**)Walking track analyses setup, (**B**) SFI test results of operated rats through 6 weeks of follow-ups, (**C**) SSI test results of operated rats through 6 weeks of follow-ups. Group 1: control, group 2: non-conductive conduit without electrical stimulation (ES), group 3: conductive scaffold without ES, group 4: non-conductive scaffold with ES, and group 5: conductive scaffold with ES.

The SFI test was performed to assess the functional recovery of the hindlimb. Figure 5 shows the setup, indexes, and their measures for SFI and SSI calculations. One-way ANOVA showed a significant difference between groups at weeks 4 and 6. Post-hoc tests demonstrated that at 6 weeks, group 5 (conductive + ES) had significantly higher SFI scores than groups 1 (control), 2 (non-conductive − ES), and 3 (conductive − ES) (all p-values were under 0.01); the comparisons between other groups were insignificant. In week 4, the post-hoc test demonstrated that only the difference between group 5 and group 2 was significant. Moreover, trend analysis using a post-hoc Tukey test on a fitted linear-mixed model demonstrated that groups 5, 4, 2, and 1 had higher scores in week 6 compared to week 2 (*p* < 0.001, *p* < 0.001, *p* < 0.05, and *p* < 0.05, respectively). Additionally, groups 5 and 4 had higher SFI scores at week 4 compared to week 2 (*p* < 0.001 and *p* < 0.01, respectively).

#### SSI

The SSI test was used to assess the static sciatic recovery by exempting the print length of the paw (Fig. 4C). Post-hoc analysis demonstrated significantly higher SSI scores at week 4 in groups 5 (conductive + ES) and 4 (non-conductive + ES) compared to group 2 (non-conductive − ES) (both had *p* < 0.01). At week 6, it was demonstrated that group 5 had higher scores compared to groups 2 and 3 (conductive − ES) (*p* < 0.05 and *p* < 0.01, respectively). Also, group 4 had higher SSI compared to group 3 (*p* < 0.05).

### Histopathological examination

#### Assessment of scaffold biocompatibility

The local tissue response post-implantation was evaluated by observing inflammation, necrosis, edema, and congestion during the early and late stages of healing, precisely 2- and 6-weeks post-surgery. A ubiquitous inflammatory response was noted in all rat specimens, with a notable extension of neuron necrosis to central nerve regions in non-conductive scaffolds. Peripheral infiltrates were predominantly observed in the nerve conduits during the early phase, which gradually dispersed and reduced size. Edema, attributable to the surgical intervention, was detected after the initial week but entirely resolved over time. Mild to moderate congestion was evident in non-stimulated groups while completely absent in others. Fibroplasia, excluding non-conductive, non-stimulated groups, appeared in the injured nerves during the late phase. The scaffolds maintained their structural integrity throughout the experiment, permitting cellular colonization, which incrementally increased until the study's conclusion.

#### Nerve regeneration evaluation

**Figure 6:**
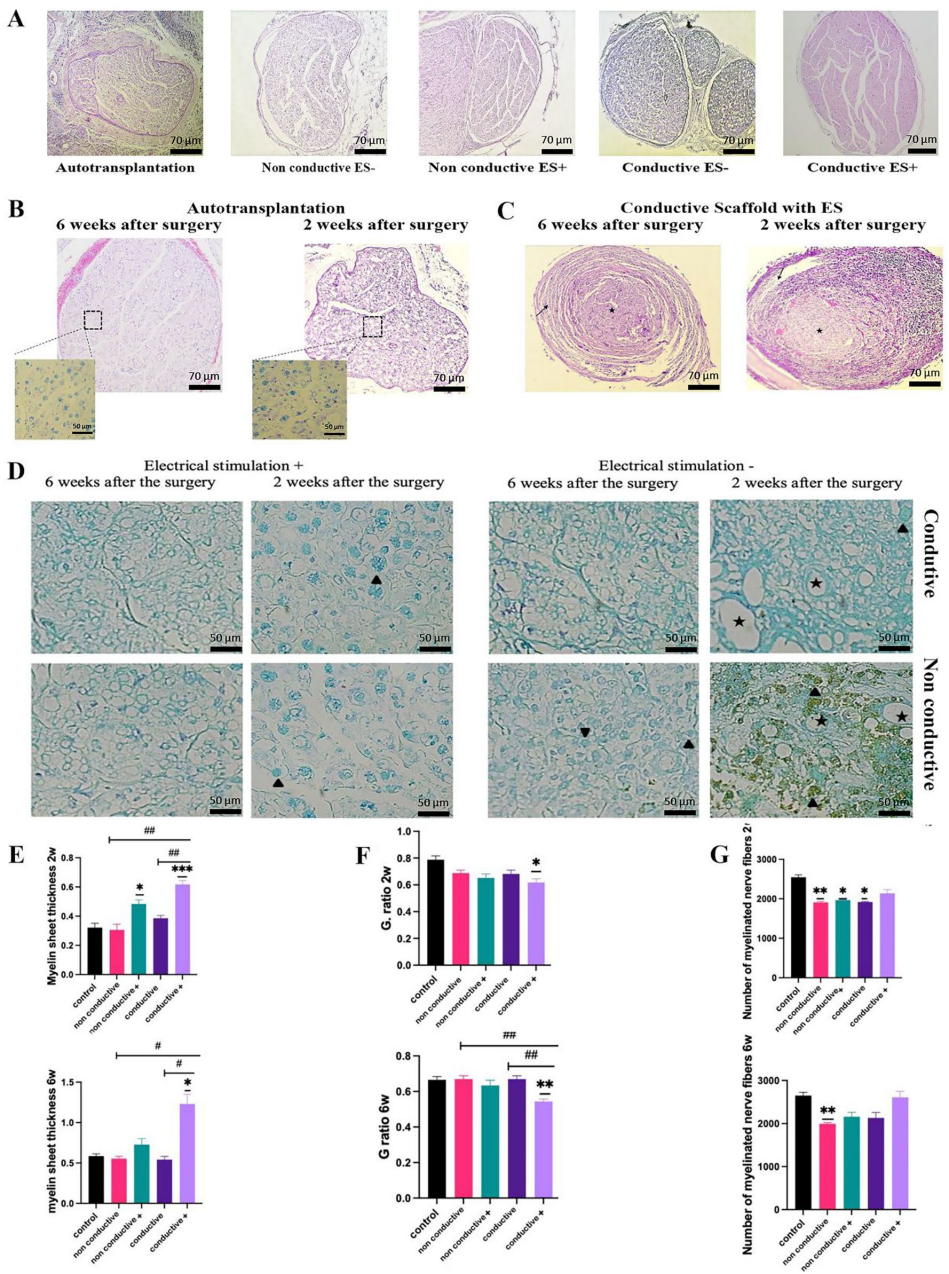
Histological evaluations. (**A**) H&E staining of autograft tissue and conduits 6 weeks after transplantation, (**B**) H&E and LFB staining of sciatic cross section after autograft transplantation two and six weeks after the surgery (×100 and ×1000). (**C**) Dynamic interactions between the sciatic nerve (asterisks) and implanted conduit (arrows) during sciatic serve regeneration. Initial observations reveal an influx of immunocytes, engorged vessels, and edema, suggesting a pronounced inflammatory response in the early stage of reconstruction. Notably, the conduit exhibits adaptability, expanding during this inflammatory phase (right). Six weeks post-implantation, inflammatory features are markedly reduced. The scaffold subsequently underwent morphological adjustments, constricting to encapsulate the nerve entirely (left) (H&E, ×100). (**D**) LFB staining of sciatic middle cross sections of conduit receiving groups, 2 and 6 post-surgery days. Various forms of myelin damage, including myelin ovoids (arrowheads) and axonal loss are seen in not electrically stimulated groups. Note swollen axons (asterisks), irregular myelin figures, and fragments, which indicate ongoing Wallerian degeneration in the non-conductive group − ES. At the same time, axonal density has a notable density in the non-conductive group + ES (×100). Analysis for myelin thickness (**E**), G-ratio (**F**), and number of myelinated nerves (**G**). * shows a comparison with the control group, and # shows the comparisons between groups. *, #: *P* < 0.05; **, ##: *P* < 0.01.

Histological analysis was utilized to evaluate sciatic nerve regeneration in the presence of conduits with and without ES over time (Fig. 6A–D). Evaluation parameters included the physical characteristics and myelination of the nerve, alongside the repopulation of the damaged area by Schwann cells and their subsequent organization into Büngner bands, crucial for axon regrowth during the extended nerve repair period. Initial assessments (week 2) revealed a disrupted arrangement of sciatic nerve fibers in the non-conductive group, as well as axonal swelling, and extensive myelin fragmentation, indicative of varying stages of Wallerian degeneration in non-ES groups. Concurrently, numerous signs of nerve degeneration were identified, including irregular myelin figures, multiple autophagic Schwann cells containing myelin fragments, and myelin ovoids. Contrarily, in the conductive conduit + ES, the dominant pathological diagnosis was axonal swelling, with limited signs of progressive neural degeneration, suggesting a mild and early stage of Wallerian change. In the late evaluation phase (6 weeks), signs of ongoing Wallerian degeneration, such as vacuolation, myelin debris, and diffuse axonal loss (indicated by fibroplasia), were seen in non-conductive conduits irrespective of ES. However, conductive scaffolds + ES demonstrated minimal nerve degeneration and sporadic axon sprouting, signifying nerve regeneration. Histopathological examination of the autograft group revealed varying degrees of vacuolation, yet an acceptable arrangement of sciatic nerve fibers was maintained.

#### Histomorphometry analysis

During the initial observation phase, the autograft group was found to have the highest count of myelinated nerve fibers based on quantitative analyses. There was no notable difference between auto-transplantation and the conductive scaffold + ES. In contrast, other groups demonstrated a marked decrease in the number of myelinated axons relative to the autograft. Both electrically stimulated groups had notably thicker myelin sheets (*p* < 0.005 and *p* < 0.001, respectively, for non-conductive and conductive conduits) compared to other groups including autograft. Additionally, the G. ratio, the ratio of the inner-to-outer diameter of a myelinated axon, for conductive + ES group was markedly lower than that of the autograft and other groups (*p* < 0.005). In the late phase, all groups, barring non-conductive − ES, had myelinated axon counts surpassing 2,000, with no significant disparities observed among groups of non-conductive + ES, conductive + ES, conductive − ES and control. However, compared to the control group, group conductive + ES exhibited a significantly increased myelin sheet thickness and decreased G. ratio (*p* < 0.005 and *p* < 0.01, respectively). Figure 6 shows histomorphometry analysis after harvesting tissues for all groups.

#### Immunohistochemical and immunofluorescent evaluations

**Figure 7:**
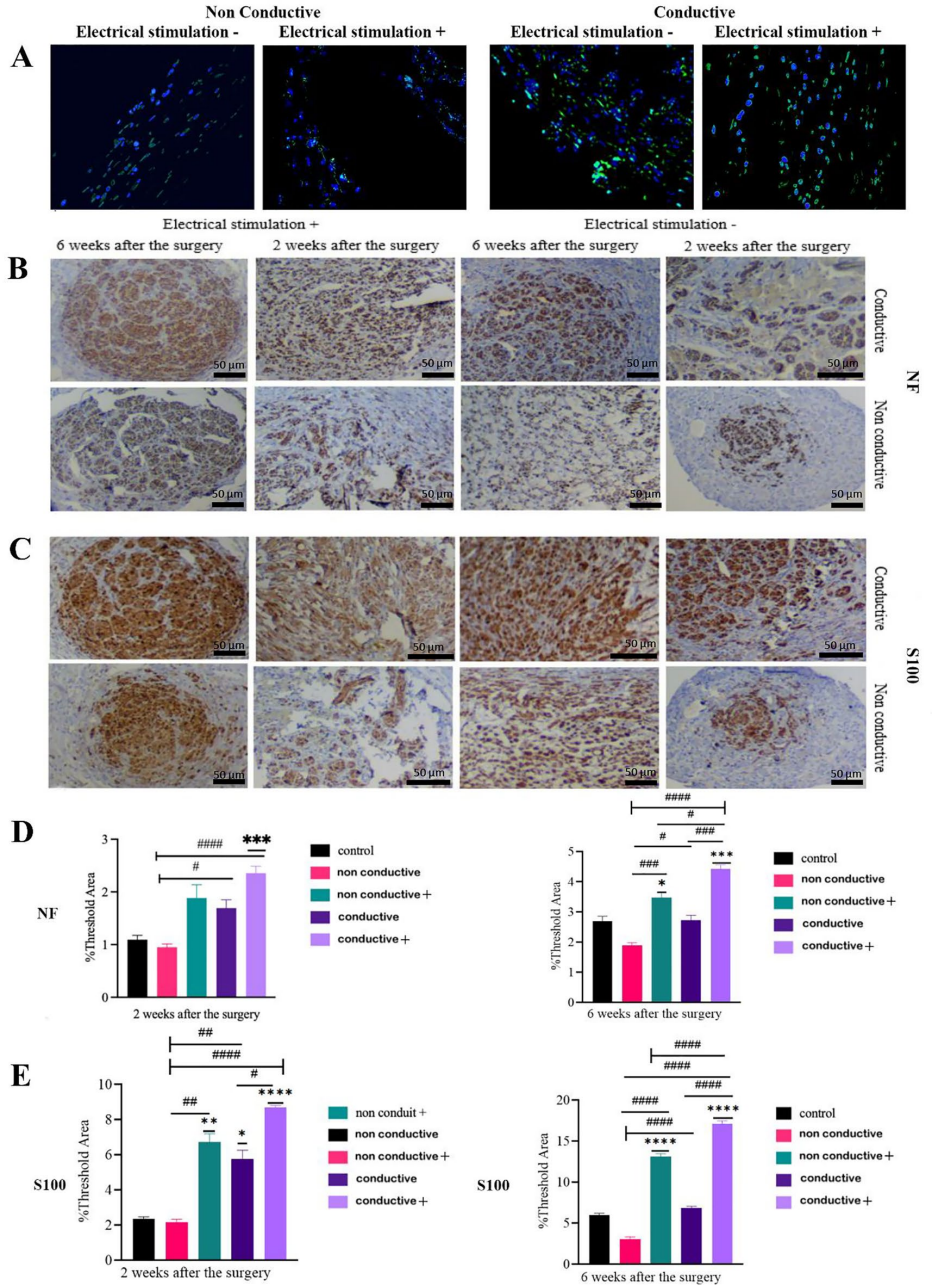
IHC and IF staining and analysis. (**A**) Green colors show the GFP positive adipose-derived mesenchymal stem cells survived 6 weeks after implantation, and blue colors are DAPI staining of the same section, showing the migration of rat cells. (**B**) IHC staining for NF and (**C**) S100. (**D**) NF and (**E**) S100 quantification based on IHC staining. * shows the comparison with the control group, and # shows the comparison between groups. *, #: *P* < 0.05; **, ##: *P* < 0.01; ***, ###: *P* < 0.001; ****, ####: *p* < 0.0001.

IHC analyses were conducted in order to quantify the regions occupied by mature axons marked by neurofilament (NF) expression and Schwann cells identified by the S100 marker (Fig. 7). The spatial distribution of these markers was meticulously examined both within and surrounding the conduit. As time progressed, the NF-stained areas in the proximal region was expanded, which reached a saturation point by the sixth week and spread into the central and distal lesion areas. IHC evaluations of neurofilaments revealed a statistically significant increase in their expression in group conductive + ES during the early phase (*p* < 0.001). During this period, the expression of S100 was notably higher in both electrically stimulated groups as well as group conductive-ES (*p* < 0.0001, *p* < 0.005, and *p* < 0.01 for group conductive + ES, conductive-ES, and non-conductive + ES, respectively). This suggests a greater presence of Schwann cells in these groups compared to the autograft group. In the subsequent phase, quantitative assessments highlighted a significant expression of these markers in both ES groups when compared with the autograft group. Additionally, immunofluorescent images showed that GFP positive ADSCs survived 6 weeks, and rat cells migrated into the scaffold after transplantation. The distribution of GFP positive ADSCs is shown in Fig. 7A. Figure 7B–E shows IHC staining and analysis for conduits.

### Ultrastructural observations

**Figure 8:**
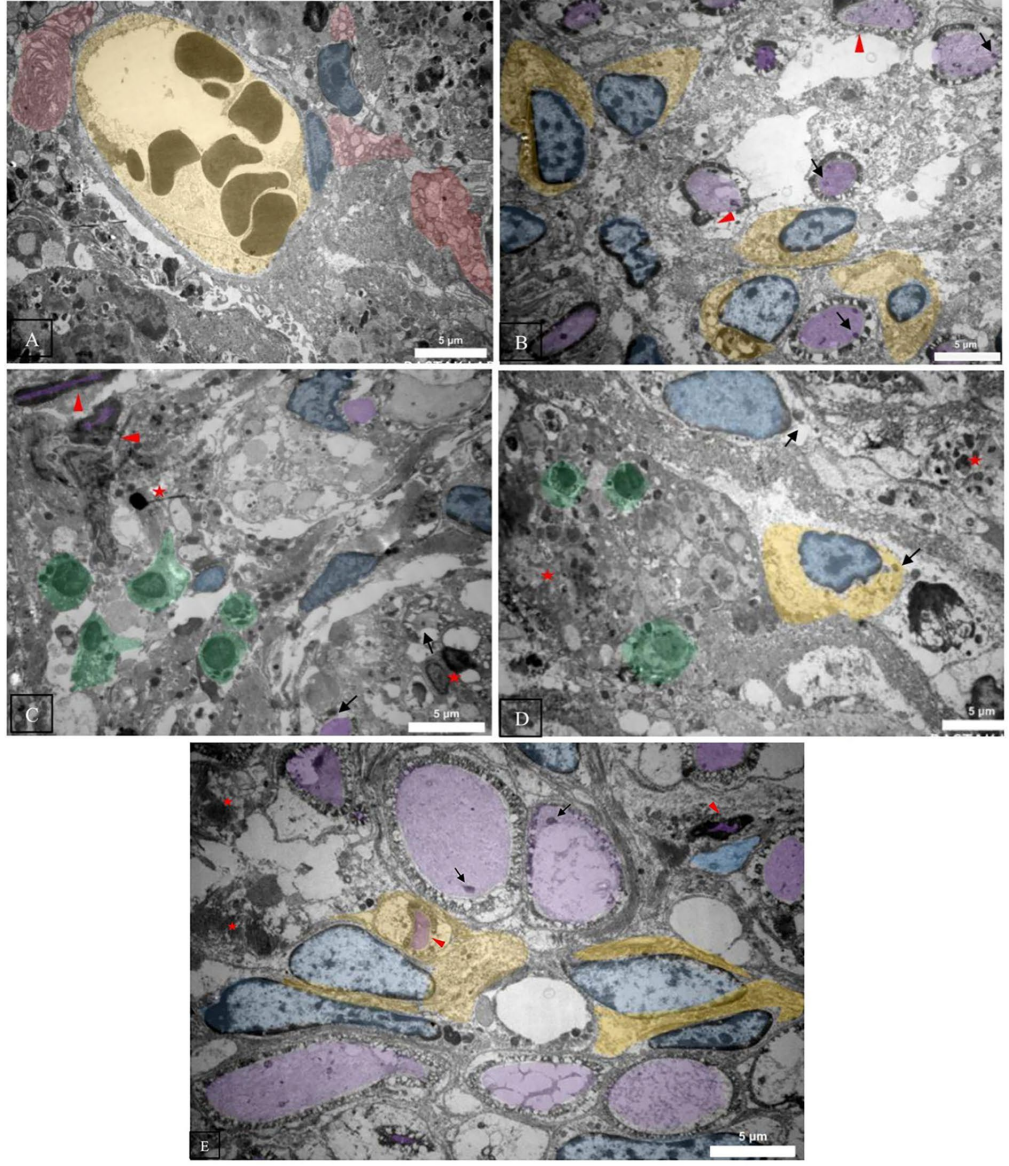
TEM images with pseudo-staining colors (**A**) In electrical stimulation, the conductive conduit promotes extensive phagocytosis of myelin debris by Schwann cells, accompanied by the noticeable presence of Büngner bands. (**B**) Conversely, when electrical stimulation is applied to a non-conductive conduit, many Schwann cells are present, but axons are mainly absent. Surviving axons exhibit neuroaxonal dystrophy with distorted myelin figures (indicated by the red arrowhead), and some axons show swollen mitochondria (indicated by the black arrow). (**C**) Without electrical stimulation with a conductive conduit, axons display irregular myelin figures and dark cytoplasm (red arrowhead). Myelin fragments (red asterisk) are scattered, and histocytes containing myelin debris are present. Swollen mitochondria are also observable (black arrow). (**D**) Without electrical stimulation and using a non-conductive conduit, fragmented myelin debris (red asterisk) and infiltration of phagocytic histocytes are significantly deposited. Injured axons exhibit numerous swollen mitochondria. (**E**) The autograft image reveals the presence of myelin fragments and debris throughout the tissue. Many axons contain swollen mitochondria, and there are limited instances of distorted myelin sheet figures (red arrowhead). Red: Büngner Bands, Blue: Schwann Cell Nuclei, Yellow: Schwann Cell Cytoplasm, Purple: Axons, Green: Histocytes.

Figure 8 shows microstructural characteristics of nerve tissues in different treatment groups. Applying ES, the conductive conduit promotes extensive phagocytosis of myelin debris by Schwann cells, accompanied by the noticeable presence of Büngner bands. On the other hand, when electricity is applied to a non-conductive conduit, Schwann cells are present, but axons are mostly missing. The axons that survive exhibit neuroaxonal dystrophy and have distorted myelin figures (indicated by the red arrowhead), while some axons have swollen mitochondria. Without ES using a conductive conduit, the axons exhibit irregular myelin figures and dark cytoplasm. Scattered fragments of myelin and histocytes that contain myelin debris can be seen. Swollen mitochondria are also visible. The tissue shows a build-up of broken fragments of myelin, along with histocytes capable of engulfing and breaking down debris. All of these are observed without any ES and using a non-conductive conduit. Additionally, the injured axons contain numerous swollen mitochondria. The myelin sheet figures appear distorted, and the tissue presents with a significant accumulation of myelin debris and fragments.

## Discussion

Although peripheral nerves have the ability to regenerate, repairing large gaps still poses a challenge. One promising approach that has gained attention involves using nerve conduits to guide regenerating axons and provide a supportive microenvironment with ECM molecules, biochemical and physical cues for axons and glial cells. Nerve conduits also protect the injured site from invading inflammatory cells and subsequent degenerative events. Furthermore, there is growing evidence to support the beneficial effects of ES on pain relief, and recently, ES has been widely used to promote nerve regeneration^28–30,47–51^.

Our study involved the development of a conductive conduit made of SF and AuNPs. The conduit possesses suitable mechanical properties for suturing during surgical procedures. We also compared the biocompatibility and regenerative potential of two types of ECM proteins as conduit fillings for cell transplantation: acellular sciatic nerve and collagen hydrogel. Decellularized tissues offer a natural microenvironment for cells to proliferate and differentiate by retaining the native ECM and bioactive molecules. They also provide mechanical support to the growing cells by preserving the three-dimensional architecture and mimicking natural tissue structure, however, they have some limitations. For example, pore size and porosity of decellularized tissue need more modifications. Cryopreservation has emerged as a potential method to modify the microstructure of tissues, resulting in increased pore size and altered elastic modulus of the ECM. Previous studies have shown that cryopreservation can upregulate genes associated with cell migration, cell–matrix adhesion, ECM secretion, and protease activity^52^. Consistent with these findings, our study demonstrated enhanced cell proliferation within collagen hydrogel compared to decellularized sciatic nerve. This can be attributed to the larger pore size and porosity of collagen hydrogel. Immunofluorescence imaging further confirmed that collagen hydrogel provided a favorable microenvironment for both transplanted and host cells. Notably, our study showed that transplanted ADSCs remained viable for six weeks and host cells successfully migrated into the scaffold (Fig. 7A). This is a significant finding, as a previous study combining human neural progenitor cells (hNPC) with conductive conduits and ES resulted in rapid cell death after only 7 days of implantation^30^.

Furthermore, the ultrastructural observation of nerve tissues in different treatment groups revealed the repopulation and reactivation of Schwann cells at the injury site, particularly in animals treated with ES. Schwann cells play a critical role in the regeneration of peripheral nerves following injury. They promote nerve regeneration by producing growth factors and ECM molecules, forming Büngner Bands, clearing debris, promoting new blood vessels, and differentiating into repair cells^53,54^. In TEM images from the conductive conduit and ES group, the phagocytic Schwann cells and Büngner Bands were clearly visible, indicating the reactivation of Schwann cells (Fig. 8A). Numerous Schwann cells were also detected in the non-conductive and ES group. In contrast, they were absent in both groups without ES, where histocytes and swollen mitochondria were observed, indicating ongoing Wallerian Degeneration.

Additionally, the expression of S100 was notably higher in both electrically stimulated groups and the conductive conduit without ES, indicating a greater presence of Schwann cells compared to the autograft group. It indicates that the microenvironment provided by the conduit has more regenerative potential compared to autograft, and the paracrine effect of transplanted ADSCs can recruit Schwann cell more efficiently than the sciatic nerve. Similarly, there is growing evidence showing that undifferentiated ADSCs can enhance peripheral nerve regeneration by secretion of neurotrophic factors such as nerve growth factor (NGF), brain-derived neurotrophic factor (BDNF), glial cell line-derived neurotrophic factor (GDNF), and ciliary neurotrophic factor (CNTF), as well as many extracellular matrix components, such as laminin, collagen and fibronectin^55–57^. These factors provide a favorable microenvironment and promote myelin formation and nerve regeneration. Furthermore, ADSCs downregulate the expression of pro-inflammatory factors and exhibit immunosuppressive and anti-inflammatory abilities which can accelerate tissue regeneration and attenuate inflammation^58,59^, providing a microenvironment that is favorable for regeneration.

Although our previous in vitro studies demonstrated that conductive scaffolds can enhance Schwann cell proliferation and neural differentiation of ADSCs^24,25,60^, our in vivo findings indicate that providing a conductive substrate alone is insufficient in the dynamic and complex environment of injured nerve tissue. The combination of a conductive substrate with ES is crucial for minimizing the process of Wallerian degeneration and enhancing the regenerative response of Schwann cells and axons. Our histological outcomes support this conclusion, as well. During the early stages of Wallerian degeneration, ES was found to have a moderating effect. At week 6, non-conductive conduits showed ongoing degeneration, while conductive scaffolds with ES exhibited minimal degeneration. Furthermore, the axon count in the ES groups was similar to the autograft group, with higher myelin thickness observed in animals treated with conductive conduits with ES compared to other groups.

Various types of conductive conduits have been investigated for peripheral nerve regeneration, including conductive polymers such as polypyrrole (PPy)^48,51,61,62^, poly(3,4-ethylenedioxythiophene) (PEDOT)^63^, polyaniline (PANI)^64^, and micro and nanoparticles such as molybdenum microparticles^29^, carboxylic graphene oxide^48^, carbon nanotube^65^, and AuNPs. However, very few studies have investigated the regenerative effect of conductive conduits in combination with both cells and ES^30^. A study by Das et al., examined Schwann cell loaded conductive conduit composed of silk and AuNPs for repairing sciatic nerve injury. They fabricated a nanobibrous scaffold using electrospinning approach and demonstrated that pre-seeding scaffold with Schwann cells result in increased muscle action potential, better motor unit potential patterns, and improved SFI scores over 18 months^50^. Electrospinning has been widely used for nerve regeneration applications due to its ability to provide a higher surface-to-volume ratio. However, evidence shows that the incorporation of AuNPs within electrospun nanofibrous scaffolds can render them prone to fragility and cracking^60^.

Solvent casting of SF + PEO solution and subsequent removal of PEO through vigorous washing which has been used in this study provided a thin flexible membrane with the potential to be roll finely. This helical scaffold embedded with AuNPs, when introduced into the inflamed environment of injured tissue, encounters the challenges of the dynamic and reactive milieu. The tissue surrounding the injury site swells due to the inflammatory response, leading to increased fluid accumulation and cellular infiltration. In many cases, scaffolds can become a limiting factor, exacerbating tissue pressure and potentially hindering the natural regenerative process. However, the helical design and inherent elasticity of the prepared silk conduit offer a distinct advantage in this context. The silk helical scaffold showcased remarkable elasticity in the inflamed environment. It expanded adaptively in response to tissue swelling attributed to inflammation, thereby mitigating potential mechanical stresses on the nascent regenerating nerve. Six weeks post-implantation, as inflammation indicators decreased, the scaffold exhibited a constriction, encapsulating the regenerating nerve. This adaptation provided consistent mechanical support and fostered an environment conducive to ongoing nerve regeneration and mechanical protection to the regenerating nerve, absorbing potential stresses (Fig. 6C). Moreover, it ensures guidance for regenerating axons, assisting in their correct alignment.

Moreover, the walking track analyses showed that ES had a more pronounced effect on functional recovery compared to the use of conductive conduit. However, it is worth noting that the use of conductive conduits enhanced the effect of ES. Animals that received ES showed significantly higher scores at week 6 compared to the gold standard. ES effectively enhances nerve regeneration, resulting in improved axon growth, myelination, and functional recovery^27,66^. Huang et al.^66^ discovered that applying ES to the sciatic nerve following treatment with a 15 mm chitosan graft enhance motor function recovery and axonal regeneration in a rat model of sciatic nerve injury. Conductive conduits enhance neural tissue mimicry and ES signal transmission. However, conductivity alone is not enough to repair without the use of an ES application^67^. This was in line with our findings that indicate conductivity alone is not sufficient to improve functional recovery. The maximum regeneration was achieved when ES was applied to a conductive scaffold.

**Figure 9:**
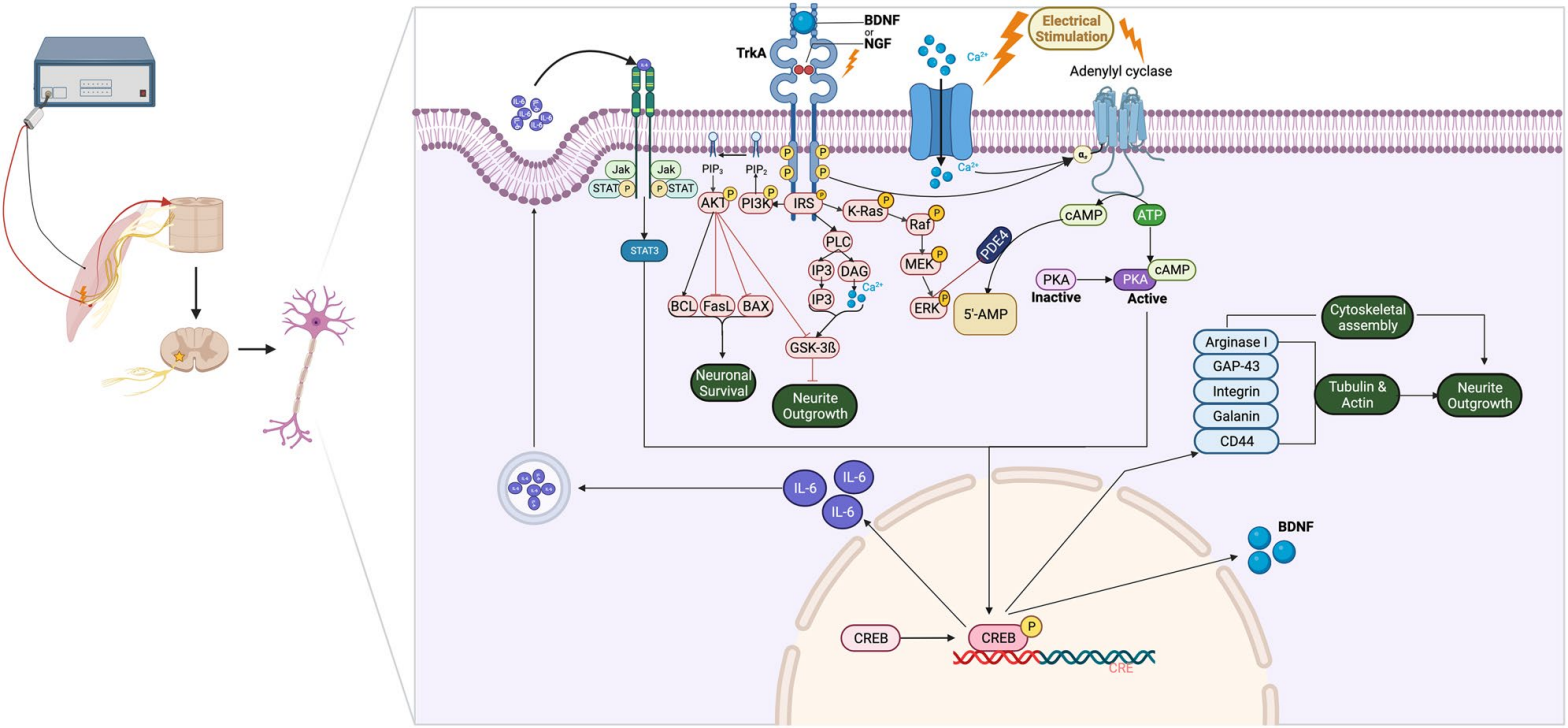
A summary of pathways involved in improved nerve regeneration following electrical stimulation application. Using ES after suturing both sides of the nerve enhances the expression of BDNF and its associated tyrosine kinase receptor by raising intracellular cAMP levels. BDNF blocks phosphodiesterase, aiding in the preservation of intracellular cAMP levels. Furthermore, elevated BDNF levels increases GAP-43 expression. The cAMP pathway triggers CREB activation, leading to enhanced expression of cytoskeletal proteins and facilitating neurite growth assembly.

Multiple mechanisms have been suggested for ES in the improvement of nerve regeneration. It has been demonstrated that the application of ES after suturing both sides of the nerve will improve the expression of the BDNF and its associated tyrosine kinase receptor through increasing intracellular cAMP. BDNF inhibits phosphodiesterase, which helps to maintain intracellular cAMP levels. Moreover, upregulation of GAP-43 has been reported in some studies, which may be a result of increased BDNF. The cAMP pathway activates CREB, increasing the expression of cytoskeletal proteins and improving assembly for neurite growth^68–71^. Figure 9 presents a summary of the pathways that are involved in the nerve regeneration induced by ES.

For the first time, we used an SF/AuNP scaffold filled with ADSC-seeded collagen and ES to regenerate sciatic nerve injury. The outcome demonstrated appropriate nerve regeneration. However, there were some limitations in our study, first of which the sample size was small and could be increased in further studies. Also, the steep learning curve of sciatic nerve injury may impact the outcome of transplantation surgery. We tried to address this issue by doing the surgery for each group at various times. Furthermore, to understand the mechanism underlying nerve regeneration, additional research could investigate the expression of regenerative factors at the treatment site.

## Conclusion

The findings from this study underscore the critical importance of combining a conductive substrate with electrical stimulation. This combination plays a pivotal role in reducing Wallerian degeneration and boosting the regenerative response of Schwann cells and axons. Our histological results further validate this conclusion. Notably, during the early phases of Wallerian degeneration, ES demonstrated a moderating effect. By week 6, non-conductive conduits exhibited ongoing degeneration, while conductive scaffolds with ES displayed minimal degeneration. Additionally, the axon count in the ES groups closely resembled that of the autograft group, with enhanced myelin thickness observed in animals treated with conductive conduits and ES compared to other groups. These findings highlight the promise of utilizing conductive substrates and ES for nerve regeneration.

## Data Availability

All data analyzed during this study are available from the corresponding author on reasonable request.
